# Assessment of pollutant load emission from combined sewer overflows based on the online monitoring

**DOI:** 10.1007/s10661-016-5461-6

**Published:** 2016-08-03

**Authors:** Agnieszka Brzezińska, Marek Zawilski, Grażyna Sakson

**Affiliations:** Institute of Environmental Engineering and Building Installations, Technical University of Lodz, Al.Politechniki 6, 90-924 Lodz, Poland

**Keywords:** Combined sewer overflow, Online sensor measurement, SOLITAX sc, UVAS plus, Data smoothing

## Abstract

Cities equipped with combined sewer systems discharge during wet weather a lot of pollutants into receiving waters by combined storm overflows (CSOs). According to the Polish legislation, CSOs should be activated no more than ten times per year, but in Lodz, most of the 18 existing CSOs operate much more frequently. To assess the pollutant load emitted by one of the existing CSOs, the sensors for measuring the concentration of total suspended solids (SOLITAX sc) and dissolved chemical oxygen demand (UVAS plus) installed in the overflow chamber as well as two flowmeters placed in the outflow sewer were used. In order to check the data from sensors, laboratory tests of combined wastewater quality were conducted simultaneously. For the analysis of the total pollutant load emitted from the overflow, the raw data was denoised using the Savitzky-Golay method. Comparing the load calculated from the analytical results to online smoothed measurements, negligible differences were found, which confirms the usefulness of applying the sensors in the combined sewer system. Online monitoring of the quantity and quality of wastewater emitted by the combined sewer overflows to water receivers, provides a considerable amount of data very useful for combined sewerage upgrading based on computer modelling, and allows for a significant reduction of laboratory analysis.

## Introduction

Due to the need to protect the water bodies, the control of both the quantity and quality of wastewater directed to a water receiver has become a necessity, in particular in case of untreated sewage from combined sewer overflows. Because significant pollution loads are emitted in this way during heavy rainfalls, the methods of exact determination as to the quantity and quality of wastewater, which largely pollutes the receivers and contributes to the deterioration of river sanitary conditions, have to be implemented. Wastewater from CSOs can result in high concentrations of suspended solids, toxic pollutants, and bacteria, especially pathogens. Additionally, a large quantity of degradable organic components may cause an oxygen deficit in the receiving waters (Walsh et al. 2005; Passerat et al. 2011; Holeton et al. 2011; Madoux-Humery et al. 2013). Potential threats to a water receiver caused by discharges of untreated sewage from a combined sewage system may be short-term, delayed, and long-term, and the impact could be hydraulic, chemical, physical, sanitary, biochemical, and aesthetic.

It is well known that the activation of the overflow depends mainly on the character of precipitation. Much research shows that there is a relation between rainfall event parameters (total depth, duration, maximum hourly and instantaneous intensity) and CSO activity. This comparison demonstrated that the total rainfall event depth was the best determinant of CSO occurrence (Schroeder et al. 2011; Yu et al. 2013, Fortier and Mailhot 2015).

During the last two decades, a rapid development of new sensor technologies has been observed. Continuous online water quality monitoring, using in-sewer sensors, has been implemented by various research groups (Häck and Lorenz 2002; Grüning and Orth 2002; Gruber et al. 2006; Hochedlinger et al. 2006a; Lacour et al. 2009). This kind of monitoring is especially effective during wet weather conditions when the quantity and quality in sewers are dynamically variable. Additionally, online measurements allow to determine pollutant loads transported in sewer systems and discharged into receivers and to analyse and simulate in-sewer processes and are very useful in real-time control (RTC) of sewer systems (Schütze et al. 2004; Campisano et al. 2013).

Due to the significant dynamics of both the quantitative and qualitative variations of wastewater flowing in a combined sewer system during rainy events, the application of the standard methods for sampling and analysing cannot give an accurate image of rapid variations of pollutant load in sewers, as well as that which is then emitted to the receivers via CSOs. The results from online measurements will also be useful as the basis for the assessment, using computer simulations, of the total load of emission from combined sewer overflows of particular subcatchments of a drainage system. The research on using online sensors for measuring the concentration of pollutants in wastewater from overflows was also carried out in Denmark, Germany, and Austria (Bechmann et al. 1999; Hochedlinger et al. 2006b; Arnbjerg-Nielsen 2011; Sandoval et al. 2013). This, in particular, confirmed their usefulness for monitoring load variations of organic substances in wastewater.

The aim of the study was to evaluate the possibility of applying online measurements of the concentration of selected wastewater pollution indicators directly in the sewer system, particularly in a storm overflow chamber, in order to fully recognize the variations in the wastewater character caused by rain events, with a particular reference to the loads emitted into the receivers.

## Materials and methods

### Study area

**Fig. 1:**
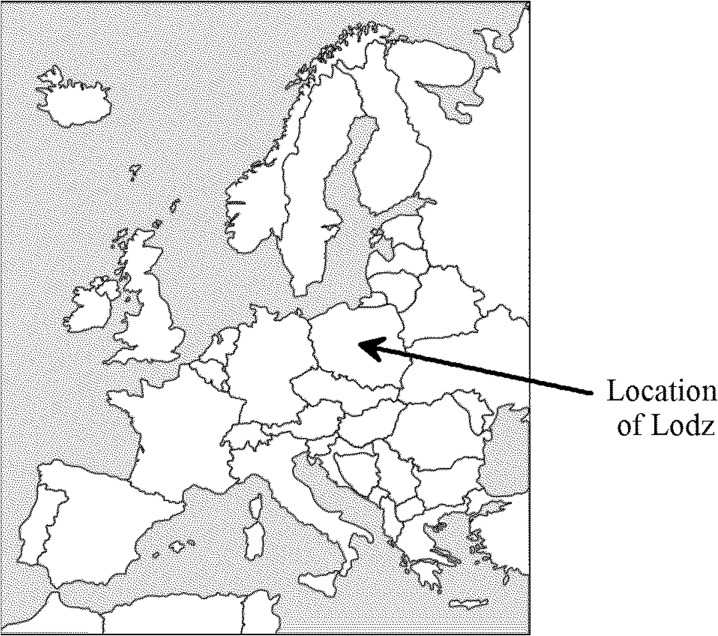
Location of the city of Lodz in Europe

The research was conducted in Lodz, which is the third largest city in Poland in terms of population (706,004) and fourth in terms of area (293.25 km^2^). Lodz is located in the central part of the country and in the middle of the Lodz Region (Fig. 1). The combined sewer system occupies an area of 43 km^2^ with a coefficient of catchment imperviousness equal to 0.43.

**Fig. 2:**
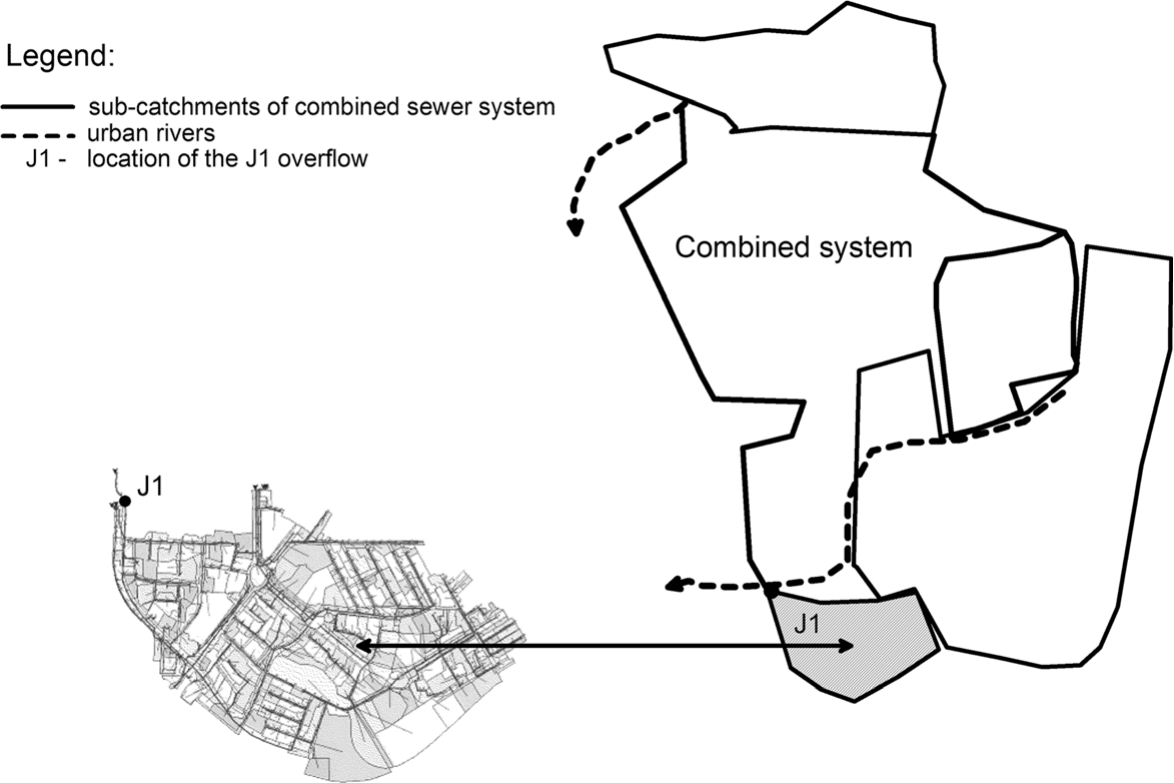
Scheme of the combined sewer system and the catchment area within the J1 overflow

The 211 ha catchment area with a multi-family residential development, connected to the examined J1 overflow, is located in the southern part of Lodz (Fig. 2). The flow of wastewater in both the outflow sewer and the overflow sewer was measured by the installed sewer flowmeters. This allows to observe the variations in the wastewater quantity during dry weather as well as to trace, in a very accurate way, the rapid variations in mixed dry flow and stormwater runoff caused by precipitation events of various character. Therefore, it gives a possibility to determine the load emitted by the combined sewer overflow accurately.

In many cases, sensors applied to concentration measurements work at the outlet from WWTPs or in the rivers where the medium is not very polluted. Sensors used in the CSO J1 were tested for 1 year at the inlet to the Group Wastewater Treatment Plant (GWWTP) in Lodz. This allowed for the observation of sensors operating in very polluted raw wastewater. The obtained results showed the need of calibration and then re-calibration of sensors after following 3–4 weeks of functioning. Re-calibration should be repeated about once a month, or depending on the need. After the 1 year test in GWWTP, sensors were installed in the chamber of CSO J1.

### Methodology of measurement

For the online analysis of the loads emitted by the combined sewer overflows, sensors from Hach Lange Company, designed to the monitoring of total suspended solids (TSS) (SOLITAX sc), and dissolved chemical oxygen demand (COD_sol_) (UVAS plus) were used. The test station consisted of two parts: the aboveground and underground one.

**Fig. 3:**
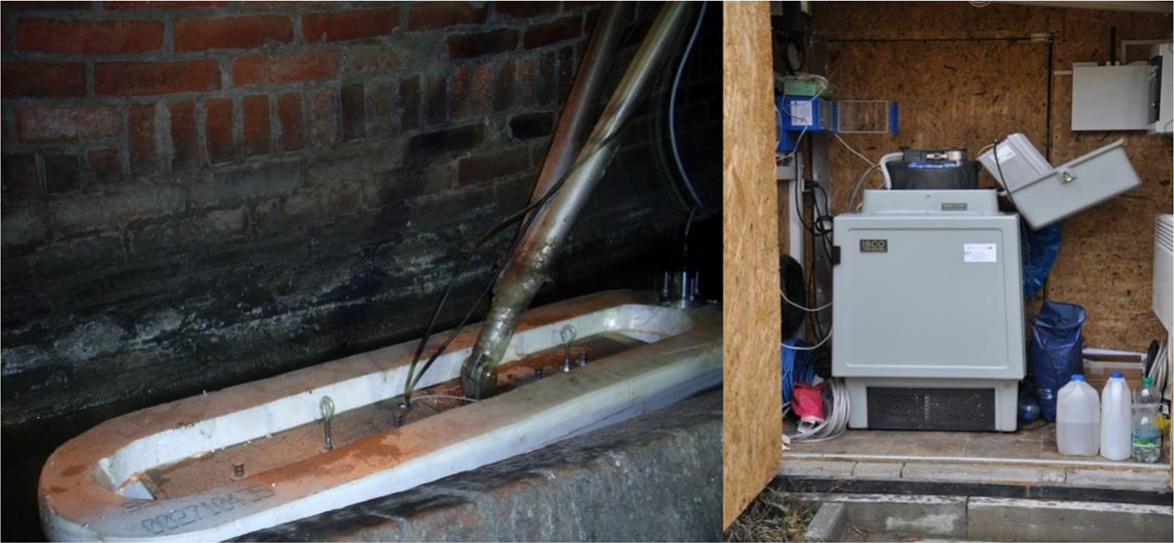
A view of the measuring position in CSO J1. **a** Pontoon with a set of online sensors. **b** Measurement box with the sampler (own photos)

The devices for measuring the concentration and the flow, together with the suction strainer collecting wastewater samples, are located inside the overflow chamber J1 and installed on the pontoon, while read-out devices and the sampler are placed in a measurement box located on the ground surface directly above the overflow (Fig. 3).

The installed online sensors use different measurement techniques (Hach information material 2015) shown in Table 1.

**Table 1:** Measurement techniques used by online sensors from the Hach (http://pl.hach.com/)

Name of the sensor	Measurement technique of sensor
SOLITAX sc	Measurement based on a combination of adsorption and diffusion of rays in the infrared according to DIN EN ISO 7027/ equivalent DIN 38414
UVASplus	Measurement of UV absorption at the wavelength of 254 nm according to DIN 38 402 C3

At the beginning of the research, the factory calibrated sensors to measure the concentration of TSS and dissolved fraction of COD (SAC254), were used. To assess the possibility of their use without frequent additional analytical tests of wastewater, the sensors have been calibrated. Test results obtained with the use of two kinds of data in several measurement series during dry and wet weather were compared. In this way, the sensors were calibrated with the average value *R*^2^ equal to 0.87 for both the dissolved COD and TSS. The calibration of these devices cannot be carried out directly on the spot. This, therefore, requires periodic inspections of online indications by conducting analytical tests, and the possible introduction of the correction to the control panel. For research, the calibration of the UVAS sensor has been adopted on soluble COD values, where the sensor indications proved to be correct during both dry and wet weather. The sensors record the data every minute, which enables a very accurate observation of variations taking place in the concentration and in the load of the examined indicators.

### Methodology of the elaboration of recorded data

The use of online sensors and flowmeters allows for the collection of a large amount of data every day, which must be verified due to possible errors in the instantaneous measurement or data transmission to the control panel.

The sensors are directly submerged in raw wastewater, so accidentally, much higher individual concentration values occur. The reason for this may be floating pollutants such as gross solids carried on the wastewater surface or directly under it, which sometimes hooked onto the pontoon or the sensors. Therefore, the data collected with the use of online sensors require adequate analysis and elimination of the erroneous recordings.

Online measurement data preparation for further analysis included the following stages:

1. Elimination of gross errors, i.e. instantaneous indications of concentration and flow equal to 0 or values much higher than those generated a few minutes earlier or later, as a consequence of false measurements (Hoppe et al. 2008).
2. The averaging of measurement results in 5-min intervals. It was verified that the arithmetic means of 5-min intervals were proven valid to the analytical values obtained from wastewater samples (therefore, the resultant total pollution load and discharge volume of wastewater obtained from the calculation of the data, with 1- and 5-min intervals, showed negligible differences).
3. Denoising the averaged data using the Savitzky-Golay method (for parameters win 20, order 2, passes 10 in case of dry weather as well as win 4, order 4, passes 4 in case of wet weather) using the Table Curve V. 5.01 software.

At the same time, laboratory tests of the wastewater quality (total suspended solids and soluble COD) emitted to a receiver from selected rainfall events were conducted.

## Discussion of results

The presented data show that the volume of discharged combined wastewater from the J1 overflow depends mainly on the depth, the intensity and the duration of precipitation. Particular rainfalls presented in Table 2 differ significantly between themselves as far as their characteristics are concerned, which influence a character of the activation of the overflow under research. Below, in Table 2, the parameters of the selected rainfalls causing the activation of wastewater overflow J1are shown.

**Table 2:** Parameters of selected rainfalls causing the overflow J1 activation during the study period

Date	*T*_dry_	*i*_mean_	*i*_max_^b^	*H*_sum_	*t*_r_	*V*_CSO_
	(days)	(mm/h)	(mm/h)	(mm)	(min)	(m^3^)
2012.03.31	120^a^	0.94	4.72	12.32	790	66
2012.04.21	21	1.34	6.42	3.70	165	663
2012.06.21	7	0.87	13.54	2.40	165	92
2012.07.14	2	3.13	14.69	3.65	70	122
2012.07.26	7	2.97	10.17	7.44	150	274
2012.08.09	2.5	5.52	14.45	7.36	80	751
2012.08.20	9	5.65	17.61	4.71	50	533
2013.08.09	8	4.6	15.6	30.1	395	7037
2013.08.10	0.2	2.4	7.2	7.6	190	682
2014. 04.08	16	1.94	20.4	5.5	170	29
2014.11.24	6	1.8	6.0	5.1	170	248
2015.05.06	1.6	1.31	13.4	12.00	552	1268
2015.05.26	0.6	1.18	19.27	9.40	480	189
2015.06.12	2.8	5.67	41.11	12.29	132	5695

^a^The first rainfall after the winter period

^b^During the time interval of 5 min

Total rainfall event depth is the best determinant of CSO. It allows for the determination of the critical precipitation during which the CSO is activated. In the case of CSO J1, the activation starts if the rainfall depth is greater than 2.5 mm.

**Fig. 4:**
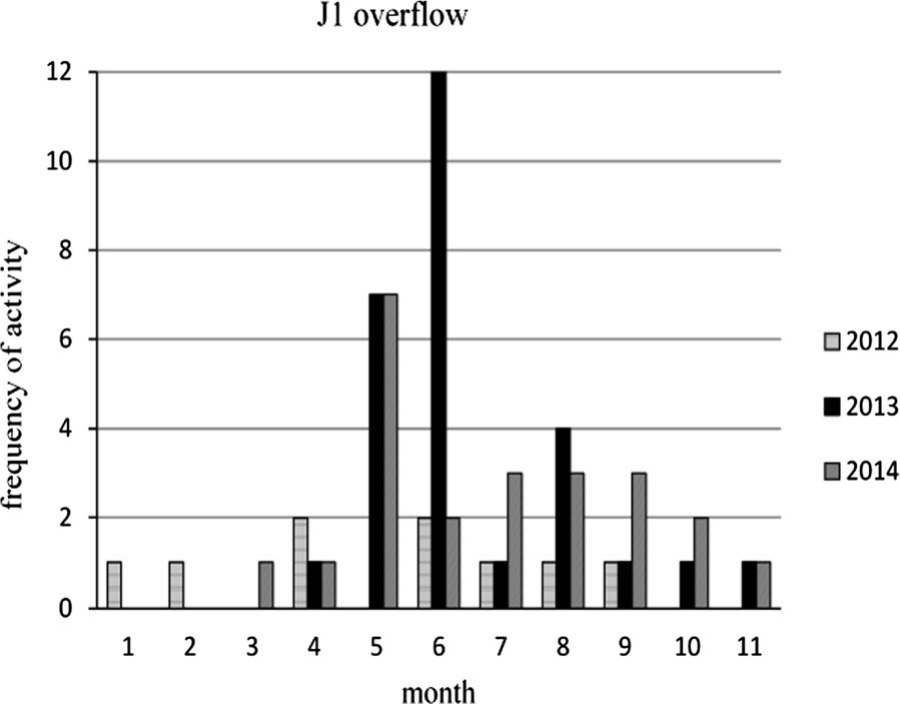
The frequency of J1 overflow activity in 2012–2014

**Fig. 5:**
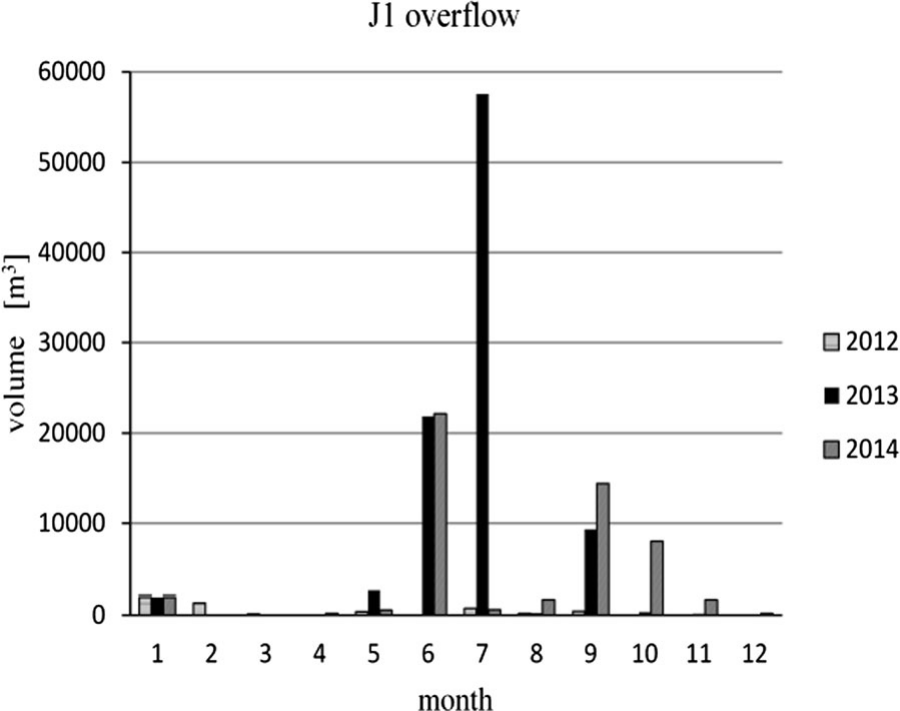
The volume of wastewater emitted by the J1 overflow in 2012–2014

In Figs. 4 and 5 the CSO J1 activity during 3 years is presented. The number of overflows was different every month and every year and varied from 9 in 2012, to 28 in 2013 and to 23 in 2014. The total amount of untreated wastewater emitted to the water receiver by this overflow during 1 year was only about 3600 m^3^ in 2012, 92,258 m^3^ in 2013, and 49,822 m^3^ in 2014. The analyses showed that the incidences of overflow exceeds those permitted by Polish legislation (up to 10 times per year) and therefore also introduces a significant load of pollutants into the river.

**Fig. 6:**
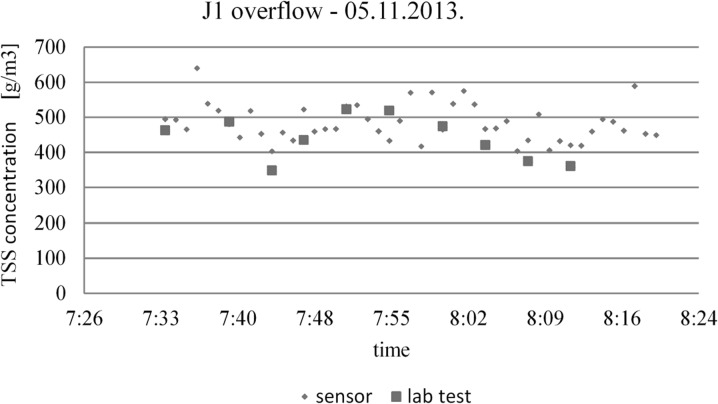
An example of a comparison between online measurement data with the lab tests for TSS

Sensors which are installed in new conditions or new media need a local calibration (Torres and Bertrand-Krajewski 2008, Gamerith et al. 2011). The calibrated sensors located in the J1 overflow chamber give very accurate values of TSS and soluble COD concentration in dry and wet weather conditions. The values obtained by online measurement figures should be very similar to the results from laboratory tests. Figure 6 shows the example of this comparison for TSS concentration during a rain event. It was observed that the differences in concentrations of this parameter between the raw measurements (without smoothing) and lab tests, were minor. Thus, it can be assumed that the load emitted from a single rainfall event, calculated by online measurement data, is very close to the reality. The use of sensors for the measurement of quality wastewater, together with a flowmeter, allows for the assessment of the pollutant load more precisely than the application of standard techniques. The conducted studies and observations have shown that the character of the pollutant concentrations run obtained from analysis and online sensor’s measurements, is very similar.

**Fig. 7:**
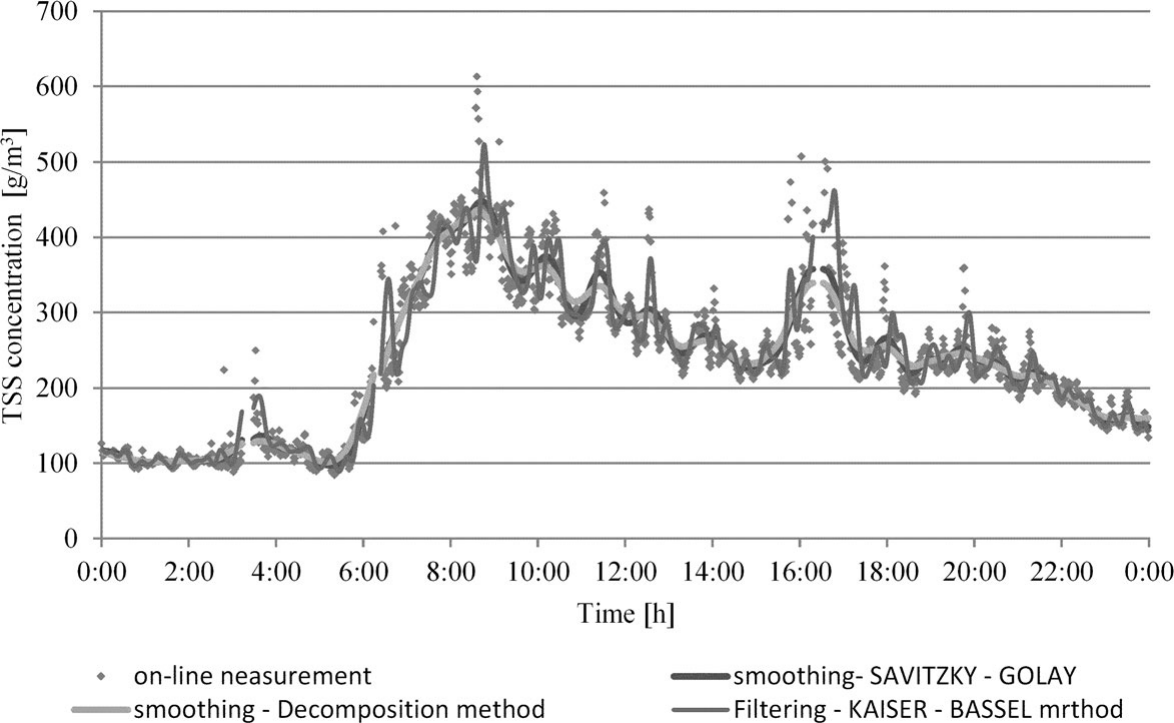
An example of run of TSS concentration smoothing on 19.03.2012 (dry weather) using different methods

Figure 7 shows the course of changes in the total suspended solids concentration according to the online measurement compared with the applied different smoothing methods and analytical results. The same was made for soluble COD.

Figure 7 shows different methods of data smoothing used for the selection of the one which best shows an adjustment to the results of the measurements. It was noticed that the finding of the superiority of the actual values (despite noticeable errors in their sensor measurements) over the values obtained by the smoothing, depends on the method used for it and its parameters (e.g. time interval, polynomial order).

**Fig. 8:**
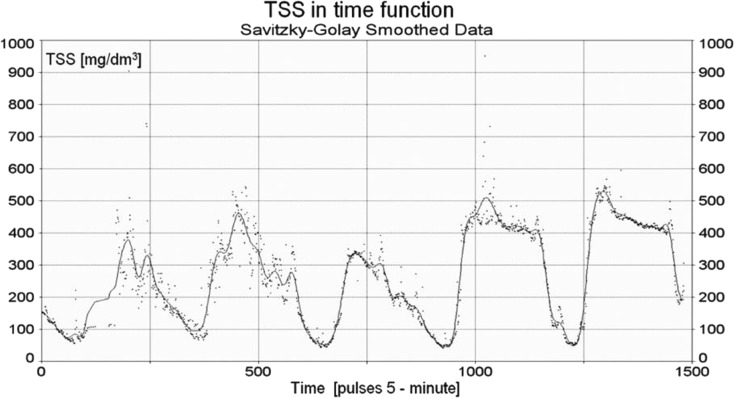
The run of raw and smoothed data of the total suspended solids concentration as a function of time

During the study, a variety of smoothing methods were used. The Savitzky and Golay filter, based on describing the smoothed values by approximating polynomial, was the most effective method (Savitzky and Golay 1964). Figure 8 illustrates an exemplary run of the total suspended solids concentration where the raw measurements from the sensor and the smoothed ones using the Savizky-Golay method were compared. The obtained total load of contaminants from the two different kinds of the presented data showed a small difference. Therefore, for the final analysis, the smoothed data was taken into account. The comparison for loads obtained using raw and denoised data showed differences less than 0.1 %. However, significant differences have been found for instantaneous values of load (as expressed in g/s), so this may be relevant in case of calibration a software for pollutant transport modelling.

The data obtained in 1-min time-steps, averaged in 5-min intervals and smoothed using the Savitzky-Golay method was the basis for the calculation of the loads emitted by the overflows to the receiver during the rainwater events (compared with the results of the laboratory analysis) (Table 3). The averaging of the actual online indications at 5-min intervals for the analysis did not affect the size of the calculated loads with respect to the online measurements (after the elimination of gross errors). Reading frequency of flow and concentration, in 1-min intervals, does not fully influence the differences in the results of the total load. Of course, the differences in the instantaneous loads have been noticed, but in total loads emitted to the environment during the whole event, the differences are practically negligible, as shown in Table 3.

**Table 3:** Comparison of the pollutant loads, for selected storm events, emitted by the overflow J1 in study period (own research)

Date	Load of total suspended solids (kg)		Load of soluble COD (kg)	
	Analytics	Sensor	Analytics	Sensor
2012.03.31	38.5	36.4	8.7	8.8
2012.04.21	412	392	88	95
2012.06.21	54	57	12.5	14.8
2012.07.14	50	52	7.7	7.9
2012.07.26	178	118	39.8	40.4
2012.08.09	402	378	53.6	49.6
2012.08.20	440	381	72	63
2013.08.09	3832	3988	581	652
2013.08.10	51	59	22	27
2014.04.08	22	27	4	5.5
2014.11.24	170	182	20	26
2015.05.06	716	765	107	123
2015.05.26	222	182	18.5	16
2015.06.12	11,012	11,196	117	112

For the quantity of load of pollution emitted into the environment, aside from the character of the rain event and the volume of discharged wastewater, the time of dry weather between rainfall responsible for the pollutants accumulation in the catchment area and the intensity of precipitation flushing them into the sewer, is also important. As it has been noted, the TSS load emitted into the receiver based on lab tests, varied during particular rain events from 22 to 11,012 kg. The range of variations of the character of the rain event was taken into account. The range of changes according to online data measurement fluctuated between 27 and 11,196 kg. Adequately, the range of the COD load was 4–581 kg using lab tests and 5.5–652 kg for the data sensor.

**Fig. 9:**
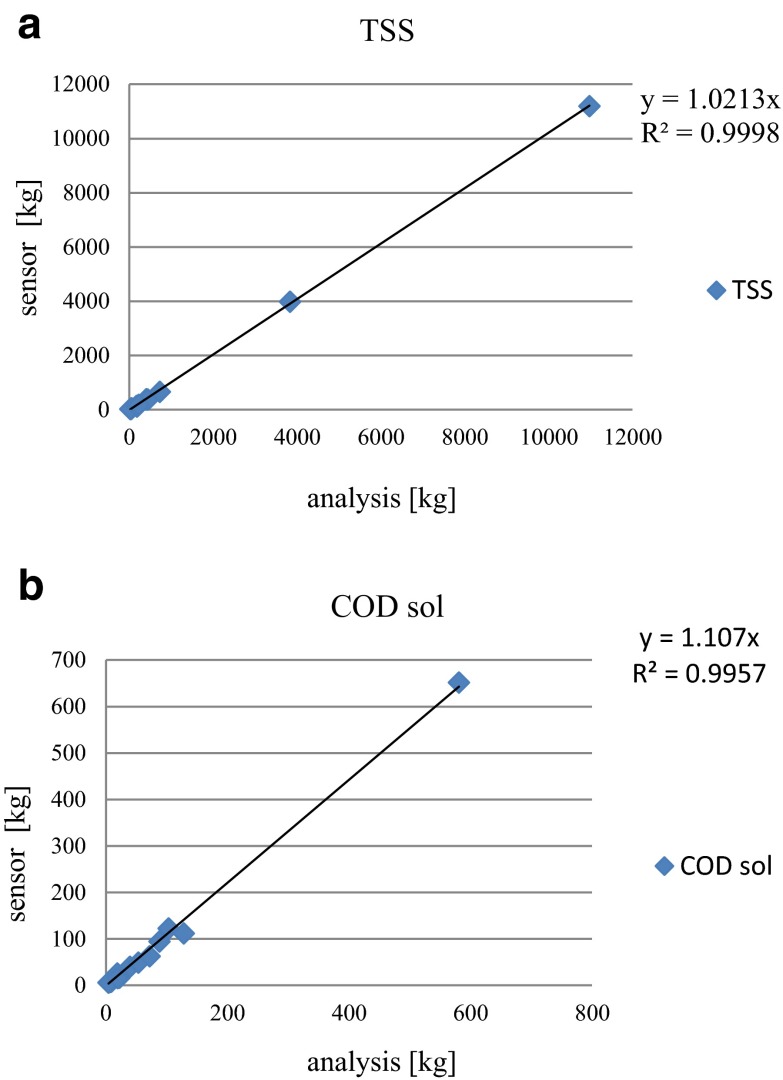
Comparison of pollutant load obtained from the analytical tests with online measurement data emitted via J1 overflow during research **a** TSS and **b** COD soluble

Despite the obtained differences in the quality of wastewater, it can be concluded that the sensors located inside the sewer system to assess the dynamics of wastewater quality, and especially, to determine the amount of the emitted load, are a good solution. The determined coefficient is very high (near 1.0) (Fig. 9), and it is obvious that regarding the load, this value can be assumed as credible.

One of the other benefits that may be achieved using sensors in the sewage system is determination of hourly variation of coefficients for concentration and flow.

**Fig. 10:**
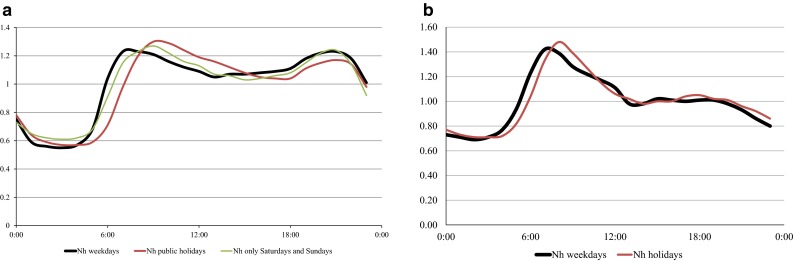
Example of the hourly variation coefficient (*N*_h_) for dry weather: **a** flow and **b** concentration of soluble COD

The online results derived from overflow chamber J1 made it possible to establish the hourly baseline pattern (*N*_h_ values) for the wastewater flow and the pollutant concentration during dry weather in this part of the sewer network. This is the background for the assessment of the dynamic changes in the wastewater composition taking place during rain events (Fig. 10).

In the graph above (Fig. 10), the differences in the characteristics of wastewater runoff between a working week, a weekend, and public holidays, are clearly noted. Such an accurate determination of the time pattern of hourly variation of flow and pollutant concentration would not be possible without the results obtained through the measurements of the sensors submerged directly in the tested medium.

The diversity of the rain event patterns presents a big problem in comparing the distribution of concentration and loads of pollutants discharged via the combined sewer overflows. Every rain event is characterized by the different dynamics of its run, a different total depth, and hence the total volume and the quality of wastewater discharged into the environment. This confirms the desirability of the use of these devices for recognizing such dynamic variations.

The conducted studies allow to come to the general conclusion that the operation of storm overflows is closely linked, not only to the character of precipitation but also to the time of day, in which there is an increased flow of wastewater into the sewage system. The same rainfall occurring at night can be entirely transported to the wastewater treatment plant, whereas during a midday dry weather peak, an excess part of rainfall must cause an overflow of polluted wastewater directed to the receiver. The collected data demonstrates how a wide range of concentration variations can occur during a single activation of an overflow which, without the use of the sensors, would be practically impossible to catch. Regarding Lodz, 15 active combined sewer overflows can be activated, and their frequency is generally higher than allowed by the national regulations (10 times per year). The overflows can be active simultaneously or individually due to the spatial distribution of the precipitation over the catchment area, but usually a couple of them are active at the same time. Therefore, the pollution load of untreated wastewater driven by small urban rivers into the river Ner, the main receiver, is large. Due to the different sensitivity of the receivers to the pollution, it would be advisable to monitor the instantaneous loads, which can be done in practice through the use of online sensors only. The decision to apply online sensors in the combined sewer system allows for a significant reduction in the cost analysis of the basic indicators of wastewater pollution and the cost of collection and transport of samples (Bertrand-Krajewski et al. 2003).

## Conclusions

The obtained data regarding the frequency of activity and the wastewater quality transported directly by CSOs to the receiver allows to take actions to decrease the frequency of the functioning of these devices (Myerscough and Digman 2008). The location of sensors in overflow chamber J1 allows for the tracking of changes in the composition of wastewater during both wet and dry weather, and above all, for capturing the dynamic and variations in the time of rain runoff to the combined sewer system.

Depending on the location of installation, the use of network flowmeters and online sensors for continuous measurement of wastewater quality, by the selected pollution indicators, provides an invaluable source of information, which can be used among others to:

- Recognize the dynamics of flow changes in sewers
- Determine the pollutant load leaving by the combined overflows
- Have a more accurate calibration of the sewerage system modelling software, especially with respect to hydraulics and wastewater quality
- Observe the first flush of pollutants in the combined sewage system
- Determine the possibility to maintain and keep sewers clean

The analysis of data derived from sensors can be taken into account when considering the possibility of using the real-time control (RTC) system, which controls the operation of the sewage system in real time, especially during torrential rainfall. The use of online sensors enables (using the data transmission) transfer of the results directly to the dispatcher, and the actual observation of the changes taking place in the sewers without the need of the presence of people in the area. This also allows, in perspective, to reduce the amount of untreated wastewater directed to the receiver using the existing methods of sewer retention (Fuchs et al. 2005). Reducing the volume of the discharged wastewater is not limited to minimize the effect of the receiver’s hydraulic loading but primarily, the physical, chemical and toxic effects.
